# Transcriptional Control of Quality Differences in the Lipid-Based Cuticle Barrier in *Drosophila suzukii* and *Drosophila melanogaster*

**DOI:** 10.3389/fgene.2020.00887

**Published:** 2020-08-06

**Authors:** Yiwen Wang, Jean-Pierre Farine, Yang Yang, Jing Yang, Weina Tang, Nicole Gehring, Jean-François Ferveur, Bernard Moussian

**Affiliations:** ^1^Section Animal Genetics, Interfaculty Institute of Cell Biology, University of Tübingen, Tübingen, Germany; ^2^School of Pharmaceutical Science and Technology, Tianjin University, Tianjin, China; ^3^Centre des Sciences du Goût et de l’Alimentation, UMR-CNRS 6265, Université de Bourgogne, Dijon, France; ^4^CNRS, Inserm, Institut de Biologie Valrose, Université Côte d’Azur, Nice, France

**Keywords:** insect, cuticle, *Drosophila*, barrier, lipid

## Abstract

Cuticle barrier efficiency in insects depends largely on cuticular lipids. To learn about the evolution of cuticle barrier function, we compared the basic properties of the cuticle inward and outward barrier function in adults of the fruit flies *Drosophila suzukii* and *Drosophila melanogaster* that live on fruits sharing a similar habitat. At low air humidity, *D. suzukii* flies desiccate faster than *D. melanogaster* flies. We observed a general trend indicating that in this respect males are less robust than females in both species. Xenobiotics penetration occurs at lower temperatures in *D. suzukii* than in *D. melanogaster*. Likewise, *D. suzukii* flies are more susceptible to contact insecticides than *D. melanogaster* flies. Thus, both the inward and outward barriers of *D. suzukii* are less efficient. Consistently, *D. suzukii* flies have less cuticular hydrocarbons (CHC) that participate as key components of the cuticle barrier. Especially, the relative amounts of branched and desaturated CHCs, known to enhance desiccation resistance, show reduced levels in *D. suzukii*. Moreover, the expression of *snustorr* (*snu*) that encodes an ABC transporter involved in barrier construction and CHC externalization, is strongly suppressed in *D. suzukii*. Hence, species-specific genetic programs regulate the quality of the lipid-based cuticle barrier in these two Drosophilae. Together, we conclude that the weaker inward and outward barriers of *D. suzukii* may be partly explained by differences in CHC composition and by a reduced Snu-dependent transport rate of CHCs to the surface. In turn, this suggests that *snu* is an ecologically adjustable and therefore relevant gene in cuticle barrier efficiency.

## Introduction

Fruit flies of the genus *Drosophila* including *Drosophila*, *Sophophora*, and Hawaiian *Drosophila* (O’Grady and DeSalle, 2018b) commonly live on fruits that serve as a site for feeding, mating, oviposition, and development. Usually, despite of the common macrohabitat, i.e., orchards, *Drosophila* flies of different species may occupy their specific microhabitats and largely avoid each other in time and space (Mitsui et al., 2006; Beltrami et al., 2012; O’Grady and DeSalle, 2018a; Plantamp et al., 2019). Some species such as *Drosophila suzukii* prefer, for instance, immature fruits (Lee et al., 2011; Walsh et al., 2011; Silva-Soares et al., 2017; Olazcuaga et al., 2019), while others like *Drosophila melanogaster* prefer rotten fruits (Versace et al., 2016).

Microhabitat choice is complex and involves along with behavior and diet preference chemo-physical environmental constraints. A major challenge in microhabitat choice is availability of water including air humidity. One strategy to cope with the water problem is to adapt the efficiency of the cuticular barrier to the specific needs. Molecular mechanisms modulating a cuticle barrier against water loss (desiccation) and accounting for adaptation to different humidity conditions have been analyzed only in a few cases. The differences in desiccation resistance between the closely related East-Australian *Drosophila birchii* and *Drosophila serrata*, for instance, rely on the composition of cuticular hydrocarbons (CHCs) that serve as a barrier at the cuticle surface (Chung et al., 2014). In *D. birchii*, the expression level of *mFas* that codes for a fatty acid synthase producing methyl-branched CHCs is suppressed compared to its expression level in *D. serrata*. Thus, reduced methyl-branched CHCs may explain why *D. birchii* is more sensitive to desiccation than *D. serrata*.

Several genes involved in cuticular desiccation resistance have been identified and characterized in *D. melanogaster*. The ABC transporters Oskyddad (Osy) and Snustorr (Snu) and the extracellular protein Snustorr-snarlik (Snsl) are needed for the constitution of the cuticle surface comprising the envelope, the outermost cuticle layer and the CHC overlay (Moussian, 2010; Zuber et al., 2018; Wang et al., 2020). Mutations in *osy*, *snu*, and *snsl* cause rapid water loss and subsequent death. Likewise, RNA interference against *cyp4g1* coding for a P450 oxidative decarbonylase required for CHC production enhances desiccation sensitivity (Qiu et al., 2012). It is yet unexplored whether the expression and function of these genes is under environmental control. In a genome-wide study, they were not found to be associated with CHC profile changes in wild-type *D. melanogaster* flies (Dembeck et al., 2015).

In this work, we sought to compare the cuticle barrier efficiency in *D. melanogaster* and *D. suzukii* in order to gain more insight in the ecology and evolution of this trait in fruit flies.

## Materials and Methods

### Fly Stocks

In the summer of 2018, five *D. suzukii* wild-type flies (2 females and 3 males) were collected from cherries from a cherry tree in a private garden in Tübingen, Germany. With these five flies, we established a *D. suzukii* stock, that we named “Tübingen 2018.” In 2019, we harvested several dozens of blackberries in a private garden in Tübingen; around 40 flies eclosed from these fruits and were used to set up a new line that was called “Brombeere 2019.” A third *D. suzukii* wild-type stock was established in 2018 starting from around 20 flies eclosed from several dozens of cherries from the city Bad Wimpfen, Germany, that is about 90 km to the north of Tübingen. The wild-type *D. melanogaster* stock was established from five female and five male flies collected in a wine orchard in Dijon in 2000 (“Dijon 2000”). All flies were raised in polysterol bottles containing standard corn meal food supplied with fresh baker’s yeast. A filter paper was stuck into *D. suzukii* culture bottles. The identical laboratory environment for both species ensures comparability of the data.

### Determination of Body Water Content

Ten male or female 5-days old flies were weighed on a micro-balance before drying for 2 h at 90°C. They were weighed for a second time after drying. The difference between the fresh and the dry weighs was used to evaluate the amount of water.

### Desiccation Assay

In each assay, groups of ten six to 10 days old flies were collected on ice and incubated in a petri dish containing silica (Sigma Aldrich) at 22°C. The petri dish was sealed with parafilm during the experiment. The experiment was repeated at least three times. Control flies survive for several hours in the petri dish without silica.

### Eosin Y Penetration Assay

According to our recently published protocol (Wang et al., 2016), ten to twenty flies of each sex and species were incubated for 20 min with Eosin Y (0.5%, Sigma-Aldrich) at different temperatures. After staining, flies were washed with tap water at room temperature. Wings were cut off using micro-scissors, observed under a Leica EZ4 stereomicroscope and imaged using the in-built Leica camera and software.

### Insecticide Treatment

Ten flies of each species were incubated in vials that contained either 0, 0.05 or 0.1 μg chlorpyriphos (stock solution 1 mg/ml acetone). Lethality was recorded during 4 h of incubation at the end of which all flies died when exposed at the highest insecticide amounts. This experiment was repeated at least three times.

### Identification of Genes and Quantitative Real-Time PCR

To design qPCR primers, *D. melanogaster*, and *D. suzukii* transcript sequences were retrieved from flybase (flybase.org). Primers (Table 1) amplifying 100–120 bp were designed using the online primer3 software^1^. Total RNA was extracted (RNEasy kit, Qiagen) from five freshly eclosed male or female flies (0 to 3 h old) to prepare the cDNA template (Omniscript RT kit, Qiagen) for the qPCR reaction (FastStart Essential DNA Green Master, Roche) on a Roche Nano LightCycler. This experiment was repeated four times. Data were analyzed with the inbuilt software of Roche and Microsoft Excel. The 2*^–^*^ΔΔ^*^CT^* method was applied to calculate the gene expression levels. The transcript levels of the reference gene *RpS20* coding for ribosomal protein S20 were used to normalize gene expression.

**TABLE 1:** Sequences of primers for qPCR used in this study.

Gene	Forward primer	Reverse primer
*D. suz app*	GGTGTCGTTTCGCAGTTCAG	TGGCTTTCTTTGTTTCTTCGGT
*D. mel app*	GAAAAGAAAGTCCCTGGGCG	ATCATCGTGTTGTCGTGCAG
*D. suz osy*	GGTGTTTGGTGGCTGGTATC	TGGTCTGACTCAGCATCACC
*D. mel osy*	GCAATATGTGACCGACGATG	GCGGTACAGCAACTGTGAGA
*D. suz cyp4g1*	CATCGATGAGAACGATGTGG	TGTCGTGACCCTCGAACATA
*D. mel cyp4g1*	ATGGCCAACAGGCATTACT	TGTCGGTGGAGTGGACAATA
*D. suz desat1*	CCACTCGTGGCTTCTTCTTC	ATGGGCATCAACAGCATGTA
*D. mel desat1*	ACGTAACCTGGCTGGTGAAC	TCTTGTAGTCCCAGGGGAAG
*D. suz fas1*	AGAGGCGAGAACCACTTTCA	AGGTGGTGGACAAGAACCTG
*D. mel fas1*	CGTACGACCCCTCTGTTGAT	ACCACCTTGAGACGTCCATC
*D. suz fas2*	CTTGATCTCCGTGCTCATCA	CAAGACGGAGCAGGCTAATC
*D. mel fas2*	CAGCAACATCGAGGAGTTCA	GCTTCTGGTGGACGCTAAAG
*D. suz fas3*	AAGCTTGTTTCGCTTTGGAA	AGCTCCACCAAAACCAAATG
*D. mel fas3*	AACGGTGTGCATCATTTTGA	CAGGAGGTCTTCGTCTTTGC
*D. suz farO*	AGAAGCCGATGCTGATGAGT	ATGGATATCCGGATGGTTGA
*D. mel farO*	AGTATCCGAACGCACTTGCT	GAAGAGATGCGCCAGATAGC
*D. suz acc*	CGGGAACAGTGACATTTGTG	CTGTTCAGCTTCTCCGGAAC
*D. mel acc*	AACAACGGAGTCACCCACA	CAGGTCACAACCGATGTACG
*D. suz snu*	GCAAGAAGAAGAACGCCAAC	TGCAAGACAGCAAAGTGGTC
*D. suz snu #2*	TTCCTCATCTCCTCGGTGTG	CCAGATCACTCCAGACAGCA
*D. mel snu*	TACACCCACTTCGGGTCTTC	AGTGCCGAGTGGAAAGCTAA
*D. suz snsl*	GTGGAACTGGGTCCTCAGAA	TTTTCTCCGTGGAGGTCATC
*D. mel snsl*	TCTGGCCCGTCAACTTTATC	CACTGGTTTCTTGGCCTGAT
*D. suz rps20*	CTGCTGCACCCAAGGATATT	AGTCTTACGGGTGGTGATGC
*D. mel rps20*	TTCGCATCACCACCCGT	TTGTGGATTCTCATCTGGAA
	AAGAC	GCG

### Determination of CHCs

To extract CHs, 6-h or 5-day old flies were frozen for 5 min at −20°C, prior to the extraction procedure. For wing analysis, wings were cut off using micro-scissors. Each pair of wings was immersed for 10 min at room temperature in vials containing 20 μl of hexane. For whole fly extraction, each individual was immersed under similar conditions in 30 μl of hexane. In all cases, the solution also contained 3.33 ng/μl of C26 (*n*-hexacosane) and 3.33 ng/μl of C30 (*n*-triacontane) as internal standards. After removing the wings or the fly bodies, the extracts were stored at −20°C until analysis. All extracts were analyzed using a Varian CP3380 gas chromatograph fitted with a flame ionization detector, a CP Sil 5CB column (25 m × 0.25 mm internal diameter; 0.1 m film thickness; Agilent), and a split–splitless injector (60 ml/min split-flow; valve opening 30 s after injection) with helium as carrier gas (velocity = 50 cm/s at 120°C). The temperature program began at 120°C, ramping at 10°C/min to 140°C, then ramping at 2 C/min to 290°C, and holding for 10 min. The chemical identity of the CHCs was checked using gas chromatography-mass spectrometry system equipped with a CP Sil 5CB column (Everaerts et al., 2010). The amount (ng/insect) of each component was calculated based on the readings obtained from the internal standards. For the sake of clarity we only show the sum (in ng) of desaturated CHs (ΣDesat), the sum of linear saturated CHs (ΣLin), and the sum of methyl-branched CHs (ΣBr) and their respective percentages calculated based on the overall CHs sum (ΣCHCs).

## Results

### *Drosophila suzukii* Is More Sensitive to Dryness Than *Drosophila melanogaster*

**FIGURE 1:**
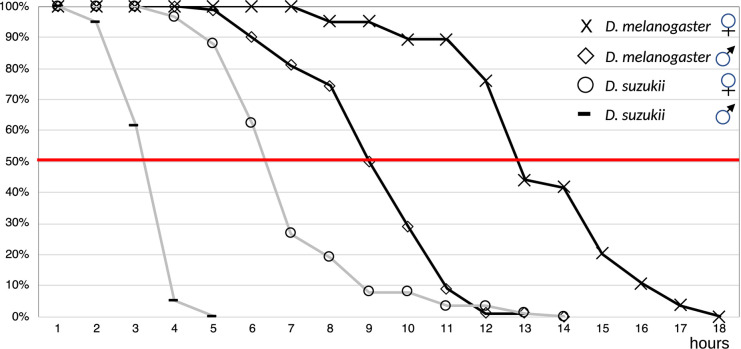
*D. suzukii* and *D. melanogaster* flies were incubated at low air humidity (<10%). Survival of flies was recorded until all flies had died. Females and males were tested separately as it is known that females are generally more robust than males. The TL50 values are 3–4 h for *D. suzukii* males, 6–7 h for *D. suzukii* females, 9–10 h for *D. melanogaster* males, and 13–14 h for *D. melanogaster* females. Following a log-rank test, the *p*-values for the differences between males and between females of both species were calculated; they are <0.0001 for both comparisons.

To learn about ecological similarities and differences between *D. suzukii* and *D. melanogaster*, we first compared their desiccation resistance (Figure 1). *D. suzukii* males were dead within 4 h after exposing them to dry conditions, while most *D. melanogaster* males survived until 5 h of exposition to dryness. They died between 6 and 8 h under this condition. *D. suzukii* females started to die after 5 h of exposure to dryness and after 10 h, their lethality reached 90%. The *D. melanogaster* females reached the 90% lethality rate after 15 h under the same condition. In conclusion, *D. suzukii* and *D. melanogaster* females live longer under dry conditions (<10% air humidity) than males. However, both *D. suzukii* males and females are more sensitive to dryness than *D. melanogaster* males and females.

### *D. suzukii* Flies Are Heavier Than *D. melanogaster* Flies but Contain a Lesser Proportion of Water

Body mass and body water content are reported to be positively correlated to desiccation resistance (Ferveur et al., 2018). We determined these two parameters in *D. suzukii* and *D. melanogaster* males and females (Table 2). *D. suzukii* males are 58% heavier than *D. melanogaster* males, while *D. suzukii* females are 75% heavier than *D. melanogaster* females. These differences are statistically significant (see Table 2). Water represents 76% and 82% of the *D. suzukii* female and male body mass, respectively, while in *D. melanogaster* females and males it represents 80% and 87%, respectively. These differences have a low statistical significance (see Table 2). Thus, *D. suzukii* flies weight more than *D. melanogaster* flies, but, by trend, contain proportionally less water.

**TABLE 2:** Body mass and water content of *D. suzukii* and *D. melanogaster* flies.

	Body mass (mg)	Water content (%)
*D. suzukii* female	1.9 ± 0.04	76 ± 0.6
*D. suzukii* male	1.2 ± 0.015	82 ± 1
*D. melanogaster* female	1.1 ± 0.09	80 ± 3.8
*D. melanogaster* male	0.76 ± 0.03	87 ± 3.1

*The *p*-values following a Student’s *t*-test for body mass mean differences between the sexes of the two species are 0.001 (females) and 0.0002 (males). The *p*-values following a Student’s *t*-test for water content mean differences between the sexes of the two species are 0.02 (females) and 0.1 (males). The standard deviation for the values of both traits are given in ±.*

### The Inward Barrier Is More Permeable in *D. suzukii* Than in *D. melanogaster*

**FIGURE 2:**
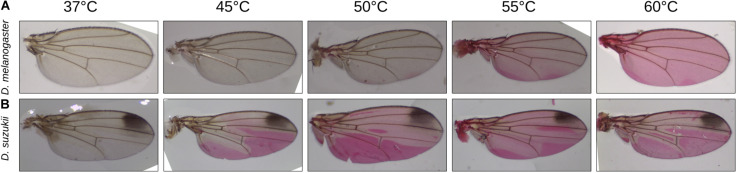
The inward barrier efficiency was tested in an Eosin Y penetration assay. As a read out, we choose to score dye penetration into the wing tissue in dependence of the incubation temperature. Here, we show the results for the male wings. Dye penetration into the wing of *D. suzukii* flies started at 40°C **(A)**, while Eosin Y penetrated the *D. melanogaster* wing at 55°C **(B)**. Penetration temperature was similar in males and females in both species. Female wings are shown in Supplementary Figure S1. It is worth mentioning that both strains were captured in the wild and are not isogenized; hence, variation of staining intensity and wing size is expected.

Our results suggest that the outward barrier is less efficient in *D. suzukii* flies than in *D. melanogaster* flies. Using an Eosin Y incubation assay (Wang et al., 2016, 2017), we tested whether the inward barrier function also differs between *D. suzukii* and *D. melanogaster* (Figure 2). In particular, we measured the permeability of the wing cuticle, given that this flat organ allows to produce an unambiguous scoring under light microscopy. The Eosin Y penetration temperature in wings lies between 37 and 40°C in both *D. suzukii* sexes, whereas its range is 55 to 60°C in both *D. melanogaster* male and female flies. Thus, the inward barrier of the *D. suzukii* wing cuticle is more permeable than the *D. melanogaster* wing cuticle.

**FIGURE 3:**
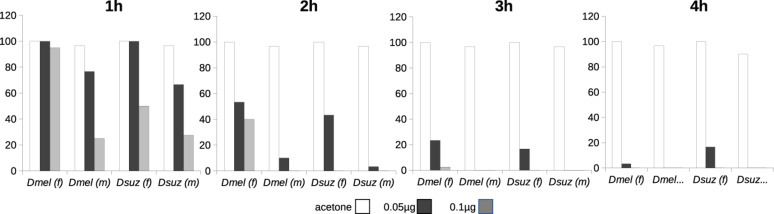
Groups of flies were exposed to three different amounts of chlorpyrifos for 4 h. Survival was recorded every hour. At least 30 flies were tested in each experiment.

To further evaluate the inward barrier efficiency, we also compared the sensitivity of *D. suzukii* and *D. melanogaster* to the contact insecticide Chlorpyrifos (Figure 3). In general, *D. suzukii* flies are more susceptible to Chlorpyrifos than *D. melanogaster* flies. We also observed that females of both species were more resistant against Chlorpyrifos than males. This finding is consistent with the assumption that the inward barrier is weaker in *D. suzukii* than in *D. melanogaster*.

### CHC Composition Differs Between *D. suzukii* and *D. melanogaster*

**FIGURE 4:**
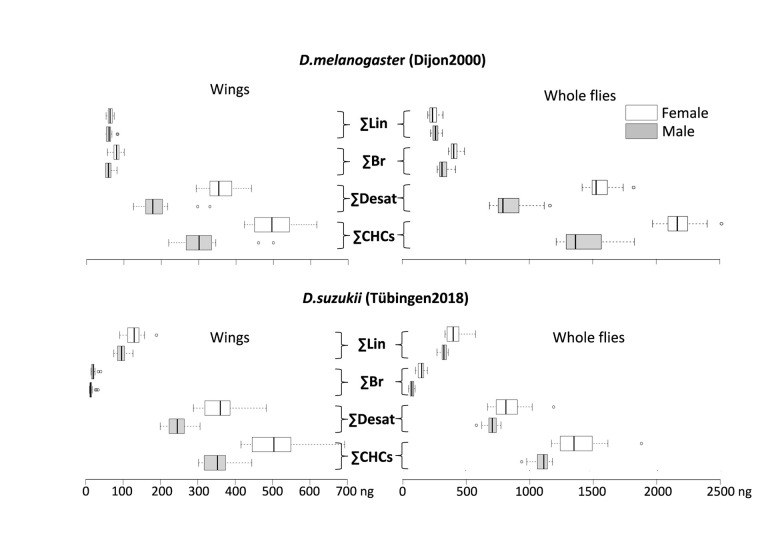
Amounts (in ng) and composition of wings (left panels) and whole body (right) CHCs in individual *D. melanogaster* **(top)** and *D. suzukii* **(bottom)** were determined by gas chromatography and mass spectrometry. The amounts of sum linear (ΣLin), sum branched (ΣBr) and sum desaturated (ΣDesat) CHCs were calculated based on the overall sum of CHCs (ΣCHCs). See Supplementary Figure S2 for all detected CHCs and Table 3 for the statistical analysis of these data.

The function of both the outward and inward barriers relies on the presence of surface CHCs. To test whether barrier differences between *D. suzukii* and *D. melanogaster* are reflected by the CHC composition, we performed GC/MS analysis of surface lipids of the whole body and of dissected wings (Figure 4). Our results are consistent with studies reporting CHC profiles in *D. suzukii* (Snellings et al., 2018) and *D. melanogaster* (Everaerts et al., 2010). On average, 1115 ng CHCs per male and 1404 ng CHCs per female were extracted from the body surface of *D. suzukii*. The body surface extracts of each *D. melanogaster* male contains 1506 ng CHCs on average, while each female *D. melanogaster* has 2095 ng CHCs on its body surface. The desaturation rates of *D. suzukii* male and female body CHCs are 63.1% and 59.1%, respectively. The desaturation rates of *D. melanogaster* male and female body CHCs are 59.3% and 71.1%, respectively. Around 6.5% of body CHCs on *D. suzukii* males and 10.4% on *D. suzukii* females are methylated. The methylation rates on *D. melanogaster* male and female body CHCs are 21.3% and 19.4%, respectively. We conclude that both the total amounts and the composition of body CHCs considerably differ between *D. suzukii* and *D. melanogaster*. In a nutshell, *D. melanogater* males and females have more branched, desaturated and total, but less linear CHCs than their *D. suzukii* counterparts. The CHC relative compositions of dissected *D. suzukii* and *D. melanogaster* wings are similar to the respective whole body CHC relative compositions.

**TABLE 3:** *p*-values of the Wilcoxon test on differences of the wing and whole body total (CHCs), desaturated (Desat), branched (Br), and linear (Lin) CHC amounts between *D. suzukii* and *D. melanogaster* males and females presented in Figure 4.

	Wings (*D. s* vs *D. m*)		Whole Body (*D. s* vs *D. m*)	
	Males	Females	Males	Females
Σ CHCs	0.021	0.837	1.907 × 10^–6^	1.907 × 10^–6^
Σ Desat	0.0007	0.588	0.0005	9.556 × 10^–5^
Σ Br	9.529 × 10^–5^	9.502 × 10^–5^	9.542 × 10^–5^	9.555 × 10^–5^
Σ Lin	9.529 × 10^–5^	9.542 × 10^–5^	0.0001	9.542 × 10^–5^

*Except for the differences of total and desaturated CHC amounts in female wings, all differences are statistically significant.*

### Expression of Key Genes Involved Barrier Formation Varies in *D. suzukii* and *D. melanogaster*

**FIGURE 5:**
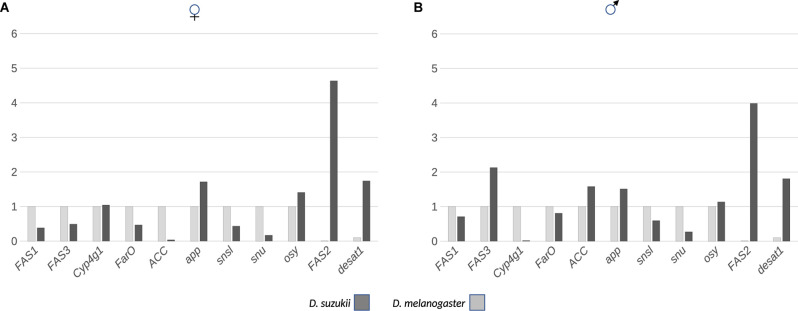
The expression of genes coding for enzymes and proteins involved in lipid (CHC) biosynthesis and transport was monitored by qPCR in females **(A)** and males **(B)** of *D. suzukii* and *D. melanogaster*. For all transcripts except for *FAS2* and *desat1*, the *D. melanogaster* expression levels were set to 1. For FAS2, the expression level of the *D. melanogaster* transcript was set to 0.01, and the expression level of the *D. melanogaster desat1* transcript was set to 0.1. The *p*-values following a Student’s *t*-test are 0.04 (female; f) and 0.33 (male; m) for *FAS1*, 0.29 (f) and 0.004 (m) for *FAS3*, 0.88 (f) and 0.004 (m) for *Cyp4G1*, 0.05 (f) and 0.048 (m) for *FarO*, 0.007 (f) and 0.04 (m) for *ACC*, 0.002 (f) and 0.046 (m) for *app*, 0.098 (f) and 0.49 (m) for *snsl*, 0.03 (f) and 0.003 (m) for *snu*, 0.047 (f) and 0.006 (m) for *osy*, 0.02 (f) and 0.0007 (m) for *FAS2* and 0.009 (f) and 0.009 (m) for *desat1* expression differences.

To learn about the molecular mechanisms underlying CHC composition in *D. suzukii* and *D. melanogaster*, we monitored the expression levels of genes that code for enzymes involved in CHC formation (Figure 5). We used quantitative real-time PCR (qPCR), to record the expression levels of *fas1*, *fas2*, *fas3*, *cyp4g1*, *farO*, *acc*, *desat1*, and *app* transcripts in newly hatched flies. Compared to *D. melanogaster* females, the expression of genes encoding the first key enzymes of fatty acid synthesis FAS1 and ACC were low in *D. suzukii* females. However, these genes showed similar expression levels in *D. melanogaster* and *D. suzukii* males. Likewise, the expression of *farO* was similar in *D. melanogaster* and *D. suzukii* males and females. While *fas3* expression was higher in *D. suzukii* males than in *D. melanogaster* males, it did not differ between *D. melanogaster* and *D. suzukii* females. The expression of the gene coding for the terminal CHC producing enzyme Cyp4g1 was lower in *D. suzukii* males as compared to *D. melanogaster* males whereas it did not differ between females of the two species. In both sexes, *app* transcript levels were higher in *D. suzukii*, while the expression levels of both *fas2* and *desat1* were around 100 times higher in *D. suzukii* than in *D. melanogaster*.

We also examined the expression levels of *oskyddad* (*osy*), *snustorr* (*snu*), and *snustorr snarlik* (*snsl*), all three coding for proteins required for the construction of the cuticle barrier (Figure 5). The expression of *snsl* did not differ significantly in both sexes of the two species. Levels of *osy* transcripts were higher in *D. suzukii* females and males than in *D. melanogaster*. However, *snu* expression was dramatically reduced in both *D. suzukii* females and males compared to same-sex *D. melanogaster* flies. We obtained identical results when we compared the expression levels of *snu* in both *D. suzukii* and *D. melanogaster* female and male dissected wing buds (Supplementary Figure S3).

Initially, *snu* was characterized in *D. melanogaster* as a key gene involved in barrier efficiency in a genetic approach in the laboratory (Zuber et al., 2018; Wang et al., 2020). Indeed, mutations in *snu* are lethal underlining its role in barrier function. Here, the quantitative variation of its expression correlating with cuticle barrier efficiency in *D. suzukii* and *D. melanogaster* suggests that it is also important in nature. To underpin this notion, we repeated the analysis of its expression changing two parameters. First, in order to rule out line-specific gene expression, we quantified *snu* transcript levels in two additional *D. suzukii* wild-type lines (see section “Materials and Methods”). Second, to exclude primer-specific effects, we designed a new set of *D. suzukii snu*-specific primers for these experiments (Table 1). The expression of *snu* was significantly reduced by more than 80% in *D. suzukii* compared to *D. melanogaster* in these experiments (Supplementary Figure S3).

Overall, the expression of some key genes coding for proteins and enzymes involved in lipid-based barrier construction and function show a substantial variation between *D. suzukii* and *D. melanogaster*. Especially, the expression divergence of *snu* is intriguing as its transcript levels have been shown to correlate with barrier efficiency in *D. melanogaster* (Wang et al., 2020).

## Discussion

### Desiccation

Desiccation resistance is conveyed by a combination of the behavioral repertoire and physiological and physical properties of the organism. In case of incipient drought, an animal, for instance, may respond by taking up and storing more water and initiate fortification of its barrier against evaporation. These responses and their amplitude depend, of course, on the genetic constitution of the organism. Hypothetically, the micro-environment of an insect has a decisive impact on the quality of a responsive trait and the expression dynamics of the underlying genes. Under identical conditions, the response may vary at several levels. In dimorphic species, a difference in this regard may prevail between sexes. Response variation is probably more accentuated between species than within species.

In our inter-species comparative approach, we found that *D. suzukii* is more sensitive to low air humidity than *D. melanogaster*. This is in agreement with a work published by Terhzaz et al. (2018). Since all flies were reared under similar laboratory conditions, we can rule out possible experimental bias for this observation. Indeed, both species were shown to be responsive to environmental cues. *D. suzukii* appears in two morphs, i.e., the summer and the winter morph with distinct ecological traits. The winter morph, for instance, is more robust at low humidity conditions than the summer morph (Toxopeus et al., 2016; Fanning et al., 2019). *D. melanogaster* as a cosmopolitan species also shows geographical variation (Rajpurohit et al., 2017; Dong et al., 2019). We believe that there are two physiological explanations for the inter-species differences. The first one implies that the difference in water shortage tolerance between these two species relies on total body water content. Indeed, we found that the overall body water content in *D. suzukii* is lower than in *D. melanogaster*. However, the water-content hypothesis is not very plausible given that *D. suzukii* males which contain relatively more water than females are less robust than females at lower air humidity. The second physiological explanation underscores that the water loss rate through the three major routes respiration, excretion or cuticular transpiration may be higher or faster in *D. suzukii* than in *D. melanogaster.* For a number of different *Drosophila* species including *D. melanogaster* (*D. suzukii* was not included in that study), it was proposed that especially cuticular transpiration accounts for the highest water loss rate, whereas excretion (<6%), and respiration (<10%) are rather negligible in this regard (Gibbs et al., 2003).

### The CHC Barrier and Gene Expression

According to the assumption of Gibbs et al. (2003), we think that a weaker outward barrier in *D. suzukii* may explain the observed difference in desiccation resistance between these species. Indeed, we found that *D. suzukii* flies have less CHCs on their surface than *D. melanogaster* flies suggesting that the CHC-based barrier is weaker in *D. suzukii*. However, Gibbs et al. (2003) reported that the quality of the cuticle barrier does not seem to depend on the CHC amounts in the *Drosophila* species studied. On the other hand, several recent works suggested that reduced CHC levels do cause enhanced desiccation (Qiu et al., 2012). Gibbs et al. (2003) also argued that the CHC melting temperature Tm that depends on CHC composition did not correlate with the water loss rate in their work. This is, however, in conflict with more recent findings. Higher proportions of desaturated CHCs confer increased desiccation resistance to *D. melanogaster* flies in experimentally selected lines (Ferveur et al., 2018). In another study, Chung et al. (2014) demonstrated that higher amounts of branched CHCs in *D. serrata* (29%) entail a higher desiccation resistance compared to *D. birchii* (3%). In analogy, our study reveals that *D. suzukii* has proportionally less branched CHCs (6% in males and 13% in females of the total amounts of CHCs) than *D. melanogaster* (23% in males and 19% in females of the total amounts of CHCs). We conclude that these traits may contribute to the difference in desiccation resistance observed between *D. suzukii* and *D. melanogaster*.

Remarkably, the CHC profiles in *D. suzukii* and *D. melanogaster* did not correlate with expression profiles of genes coding for lipid synthesis or modification enzymes. For instance, *FASN2* that codes for a microsomal fatty acid synthase catalyzing the formation of methyl-branched fatty acids, is expressed at higher levels in *D. suzukii* than in *D. melanogaster*, while the relative amounts of branched CHCs is reduced in *D. suzukii*. Moreover, the expression of *Desat1* that codes for a fatty acid desaturase responsible for fatty acid desaturation is enhanced in *D. suzukii* although the relative amounts of desaturated CHCs are lower in this species. Either the gene expression levels do not reflect the enzyme levels, or the catalytic activity of enzymes in *D. suzukii* is lower than in *D. melanogaster*. Alternatively, the limiting process of CHC amounts may not be their production but their externalization and deposition on the cuticle. Consistent with this interpretation, we found that the expression of *snu* coding for an ABC transporter involved in barrier construction and function (Zuber et al., 2018; Wang et al., 2020) is reduced in *D. suzukii* compared to *D. melanogaster*. Thus, it is possible that less CHCs accumulate on the cuticle of *D. suzukii* because of reduced Snu activity that should be tested in biochemical experiments. This interpretation is in line with our recent findings that in *D. melanogaster* flies with low *snu* expression in the wing have decreased CHC levels and compromised wing cuticle barrier function (Wang et al., 2020). Possibly, the expression divergence between *D. melanogaster* and *D. suzukii* relies on a species-specific regulatory network and involves more target genes than *snu* alone (Wittkopp, 2007). One such target gene may be *osy*, also coding for an ABC transporter needed for cuticle barrier efficiency and acting independently from Snu (Wang et al., 2020). The expression of *osy* is, in contrast to *snu*, slightly but significantly upregulated in *D. suzukii* compared to *D. melanogaster*. Obviously, this upregulation is not sufficient to equate the cuticle barrier efficiency in *D. suzukii* with the one in *D. melanogaster*. Admittedly, this is a compelling but simplified scenario as we do neglect an important point. The experiments presented in this work were done in a constant laboratory environment. Thus, we did not analyze the gene x environment (GxE) interaction in our desiccation assay. In other words, in nature, a complex, changing environment, gene expression variation may be different (Gibson, 2008; Hodgins-Davis and Townsend, 2009). For instance, the barrier of *D. suzukii* flies might be more robust under natural conditions than in the laboratory. Whether these differences may account for lifestyle differences between *D. suzukii* and *D. melanogaster* remains to be investigated.

To what extent can we apply our view on the situation of the outward barrier in *D. suzukii* and *D. melanogaster* to the situation of the inward barrier? Our experiments showed that the weaker outward barrier function of *D. suzukii* flies is paralleled by their weaker inward barrier function. Xenobiotics including both the inert dye Eosin Y and the insecticide Chlorpyrifos did penetrate the cuticle of *D. suzukii* more efficiently than in *D. melanogaster*. Thus, in a simple scenario, the CHC composition that defines the outward barrier function, would also be responsible for the inward barrier function in both species.

## Conclusion

We would like to share two concluding remarks with the reader. First, we are aware that some differences in barrier efficiency between *D. suzukii* and *D. melanogaster* may be due to some geographical differences between the cities of Tübingen (Germany) and Dijon (France), where the respective strains studied here were captured. Indeed, barrier efficiency varies among geographically separated *D. melanogaster* populations (Dong et al., 2019). *D. suzukii* populations also display genomic variations (Tait et al., 2017) that may have an impact on their desiccation tolerance. However, the difference of desiccation sensitivity between these two species is similar to that reported in a previous study indicating that *D. melanogaster* is more resistant under low air humidity than *D. suzukii* (Terhzaz et al., 2018). This confirms that inter-specific differences are higher than possible geographical intraspecific differences.

Second, here we focussed on the cuticle as a potential site of water loss during desiccation. Recently, Terhzaz et al. (2018) showed that differences in a neuroendocrine control of water loss through the Malpighian tubules, i.e., excretory water loss in *D. suzukii* and *D. melanogaster* could also explain differences in desiccation resistance. Hence, as mentioned above, besides cuticular transpiration control, other mechanisms of water homeostasis may account for performance differences between *Drosophila* species adapted to environments with variable hygrometry. Continuing work in this direction to gather the complexity of desiccation resistance or sensitivity may ultimately serve to design strategies for successful *Drosophila* pest management.

## Data Availability Statement

The raw data supporting the conclusions of this article will be made available by the authors, without undue reservation, to any qualified researcher.

## Author Contributions

YW, J-PF, YY, JY, WT, NG, J-FF, and BM performed experiments and analyzed data. J-FF and BM designed and supervised experiments. J-FF revised the manuscript. BM wrote and revised the manuscript.

## Conflict of Interest

The authors declare that the research was conducted in the absence of any commercial or financial relationships that could be construed as a potential conflict of interest.

## Footnotes

1 http://primer3.ut.ee

## Supplementary Material

FIGURE S1 As in males shown in Figure 2, Eosin Y penetrates the wings of *D. suzukii* females at lower temperatures as the wings of *D. melanogaster* females.

FIGURE S2 Here we show the array of CHCs in weight (ng) and percentage (%) as determined in wings and whole bodies of *D. suzukii* and *D. melanogaster flies*.

FIGURE S3 **(A)** As in the whole body shown in Figure 5, *snu* expression is reduced also in *D. suzukii* wings of males and females compared to *D. melanogaster*. **(B)** The expression of *snu* is reduced in three different stocks of *D. suzukii* flies (males and females mixed) compared to *D. melanogaster* flies. The *p*-values after a Student’s *t*-test are 0.0004 for the expression differences of *snu* between female wings and 0.02 for the expression differences between male wings shown in **(A)**. The *p*-values for the expression differences of *snu* between *D. melanogaster* and the different *D. suzukii* lines are 0.013 (Tübingen 2018), 0.009 (Brombeere), and 0.008 (Bad Wimpfen) shown in **(B)**.
