# Supplementary figures and images for: Transcriptional Control of Quality Differences in the Lipid-Based Cuticle Barrier in Drosophila suzukii and Drosophila melanogaster
Source: Front Genet. 2020 Aug 6;11:887. doi: 10.3389/fgene.2020.00887 (PMC7423992; doi:10.3389/fgene.2020.00887)

Supplementary figure 1

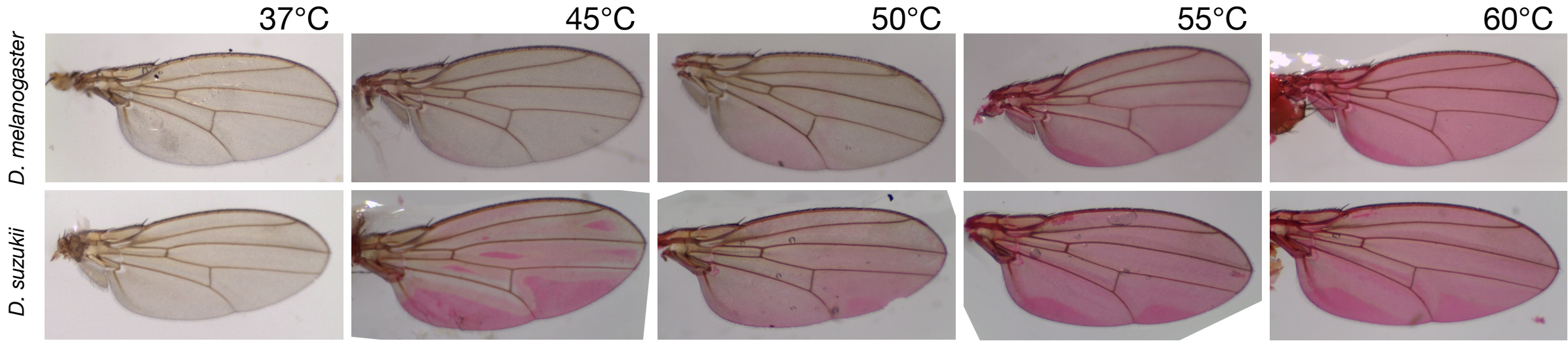

Supplement: FIGURE S1 — As in males shown in Figure 2, Eosin Y penetrates the wings of D. suzukii females at lower temperatures as the wings of D. melanogaster females. [file Data_Sheet_1.pdf]

*D. melanogaster* (Dijon 2000)

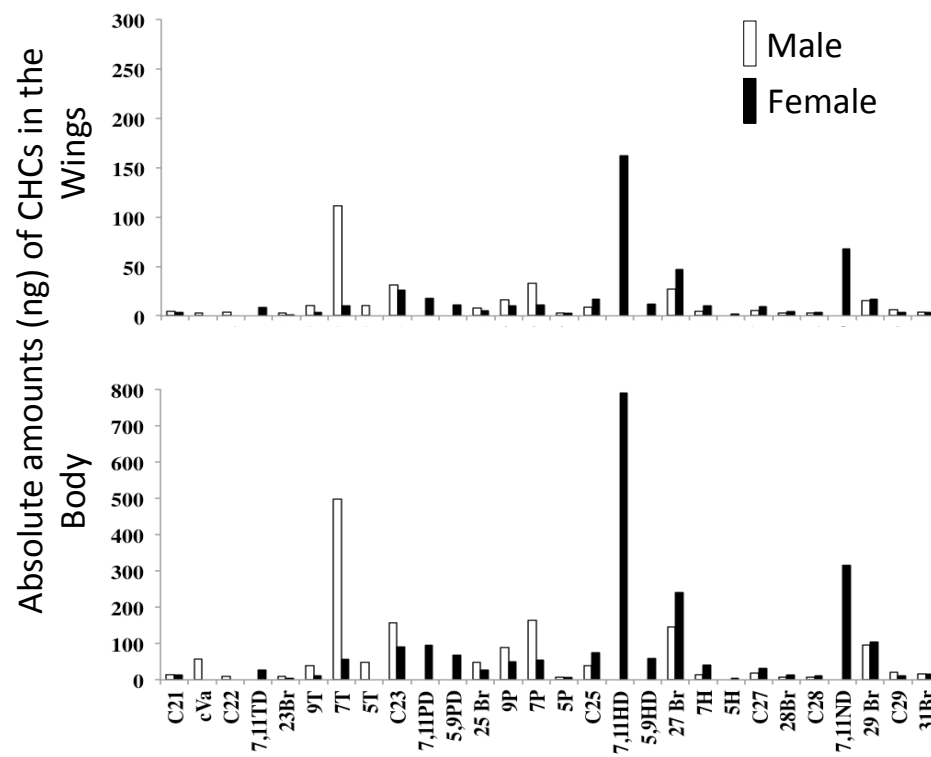

*D. sukuzii* (Tübingen 2018)

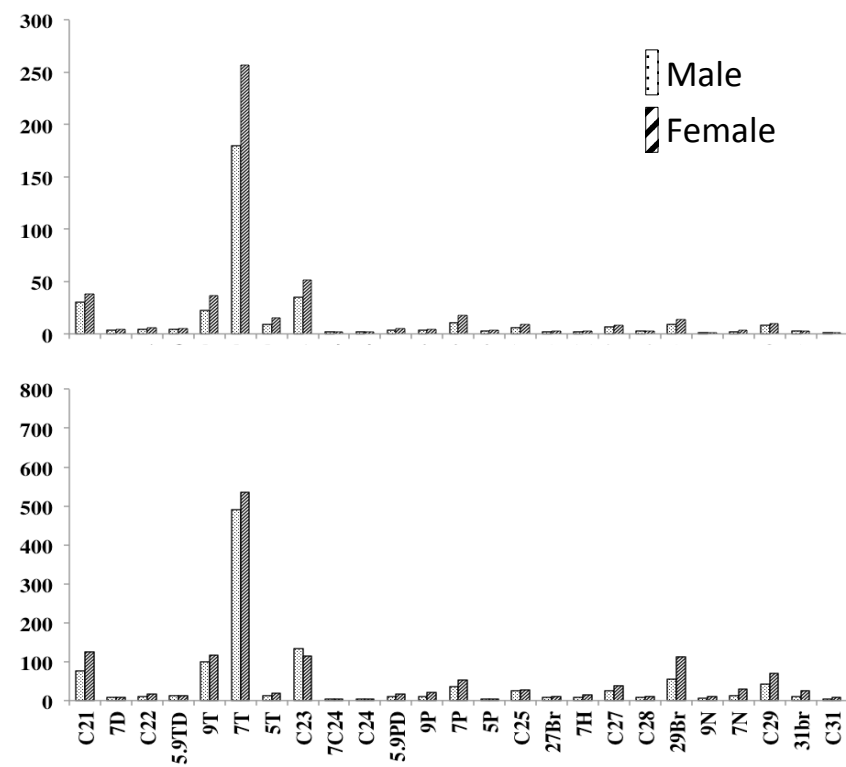

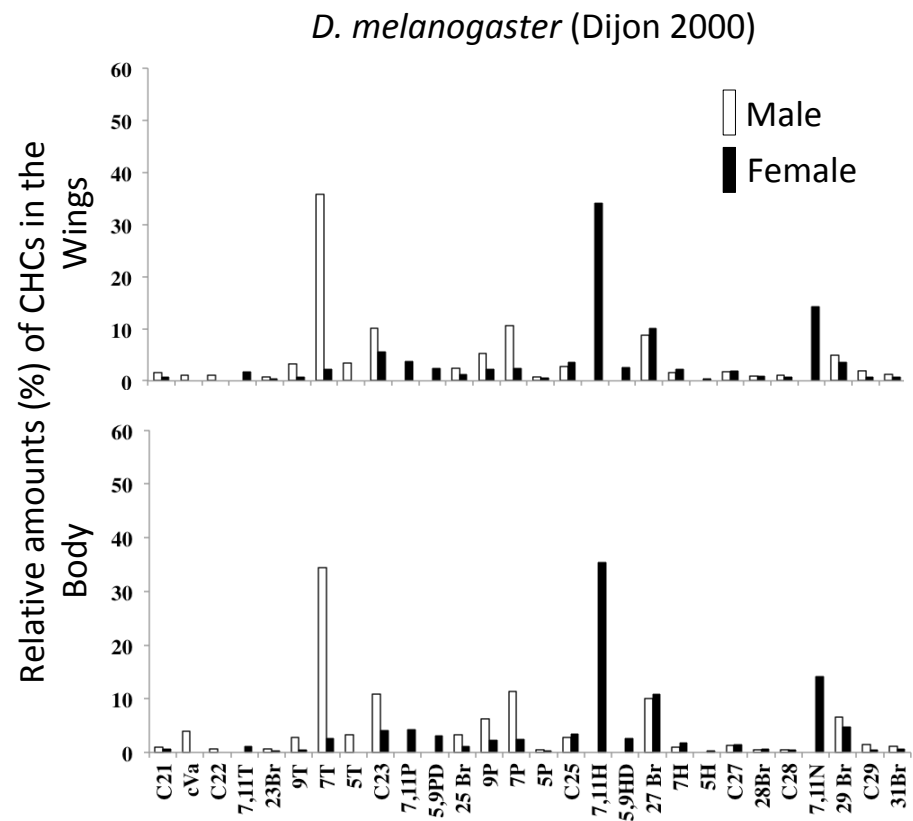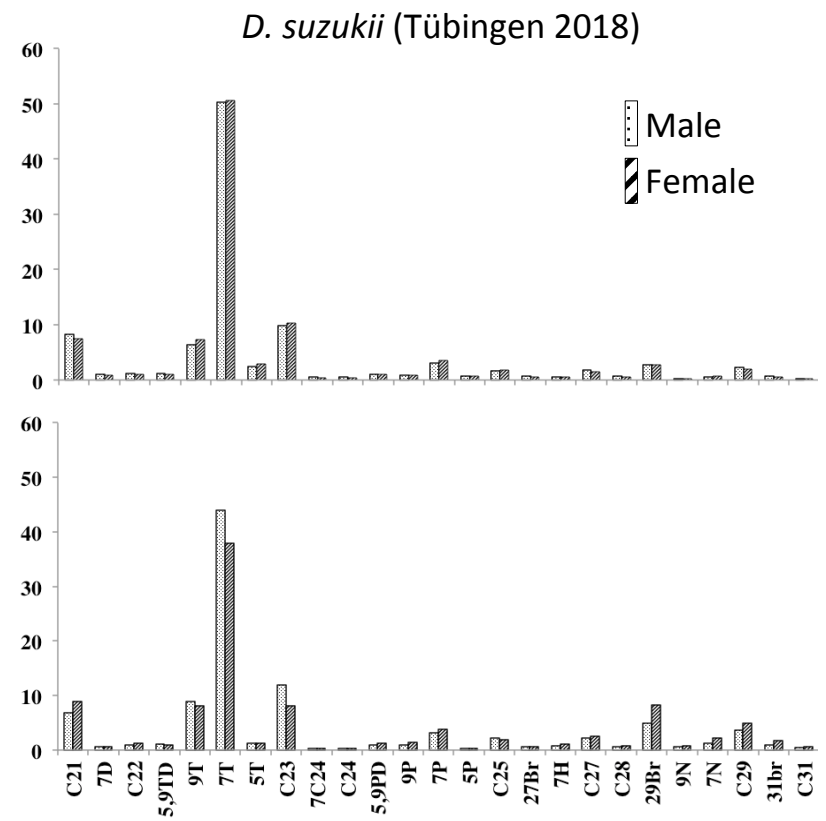

Supplement: FIGURE S2 — Here we show the array of CHCs in weight (ng) and percentage (%) as determined in wings and whole bodies of D. suzukii and D. melanogaster flies. [file Data_Sheet_2.pdf]

A

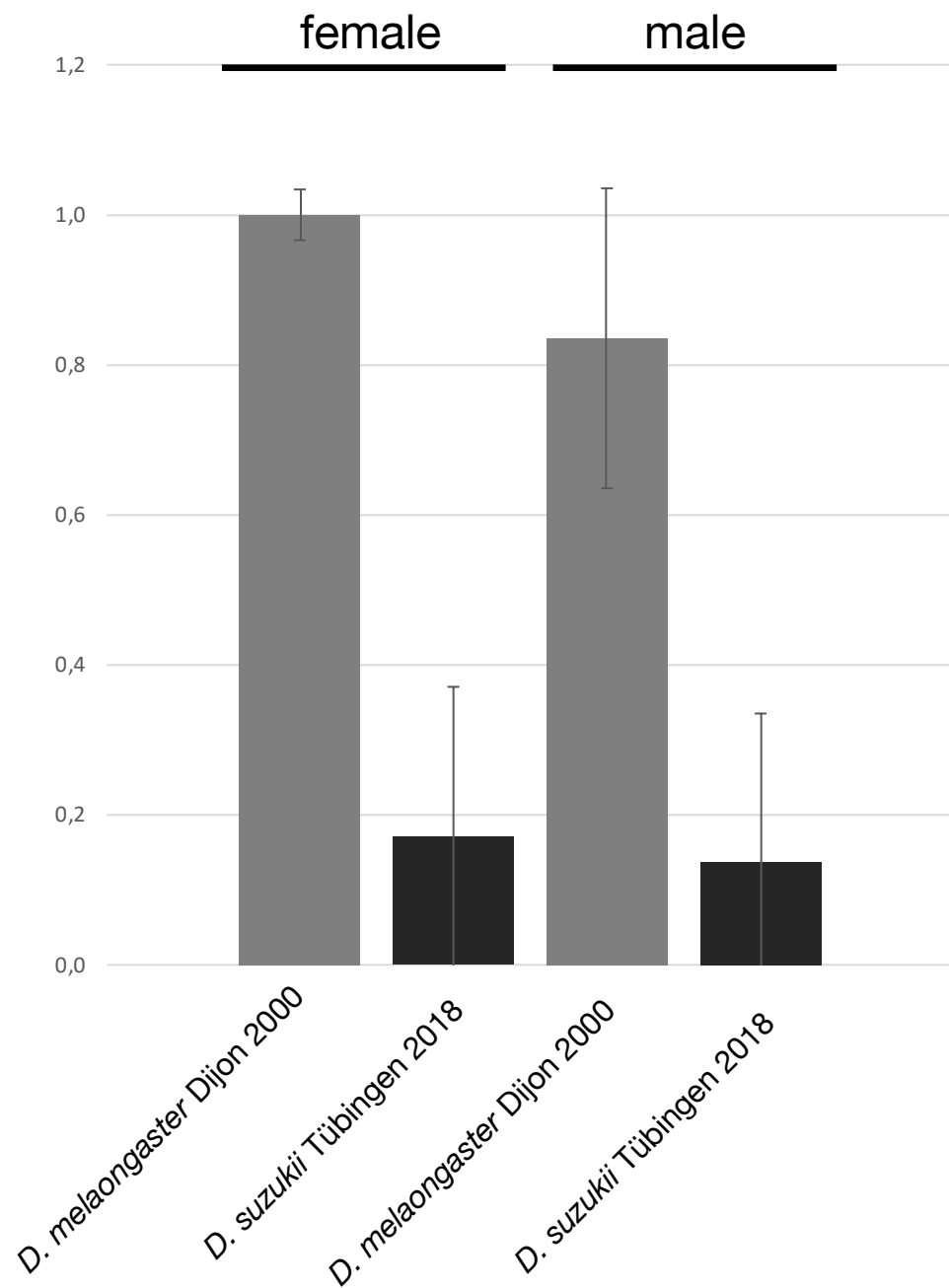

B

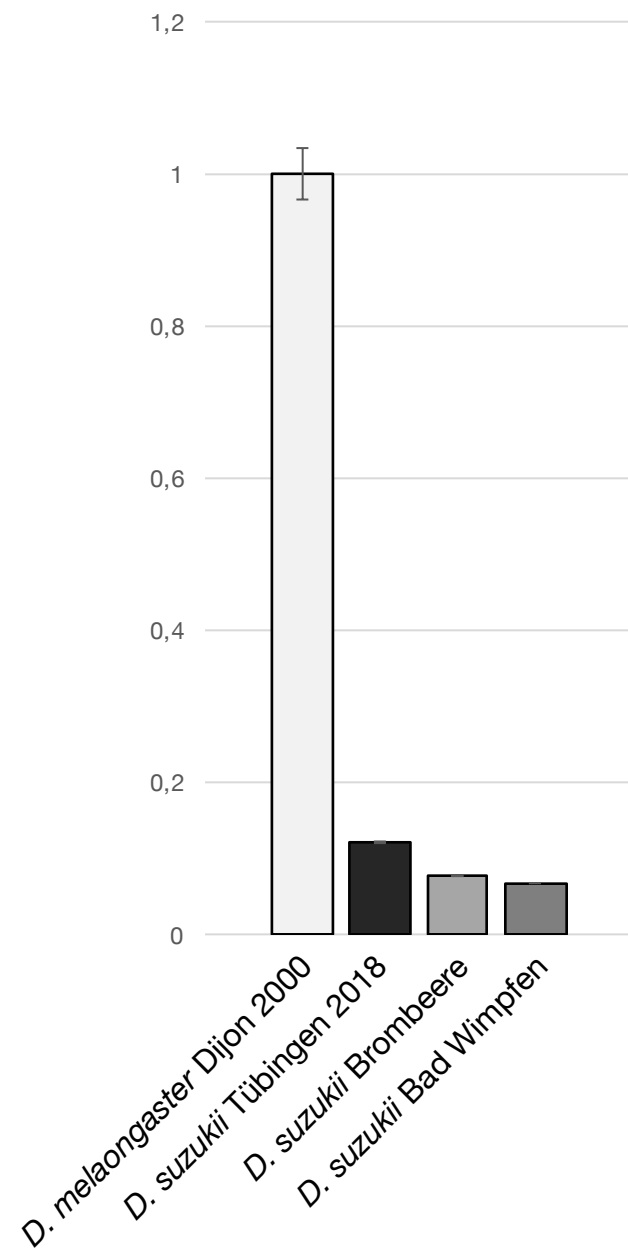

Supplement: FIGURE S3 — (A) As in the whole body shown in Figure 5, snu expression is reduced also in D. suzukii wings of males and females compared to D. melanogaster. (B) The expression of snu is reduced in three different stocks of D. suzukii flies (males and females mixed) compared to D. melanogaster flies. The p-values after a Student’s t-test are 0.0004 for the expression differences of snu between female wings and 0.02 for the expression differences between male wings shown in (A). The p-values for the expression differences of snu between D. melanogaster and the different D. suzukii lines are 0.013 (Tübingen 2018), 0.009 (Brombeere), and 0.008 (Bad Wimpfen) shown in (B). [file Data_Sheet_3.pdf]
